# Supplementary material for: Post-caesarean section infection burden, antimicrobial resistance pattern, and associated factors at all Africa Leprosy Rehabilitation and Training Center Comprehensive Specialized Hospital, Addis Ababa, Ethiopia
Source: PLoS One. 2025 Jul 31;20(7):e0326935. doi: 10.1371/journal.pone.0326935 (PMC12312969; doi:10.1371/journal.pone.0326935)
Supplement: Attachment — Submitted filename: Response to Reviewers.docx [file pone.0326935.s002.docx]

**Response to the academic editor**

1. Thank you for your feedback. We have reviewed the PLOS ONE style templates and made the necessary adjustments, including file naming conventions, and submitted the revised manuscript.

2.	We thank you for your comments. We have updated our submission to comply with PLOS LaTeX requirements to ensure proper formatting and inclusion of all necessary components in a cohesive .tex file.

3.	Thank you for pointing out the data availability statement. We have revised it to ensure compliance with the Open Data policy and will clearly outline any ethical restrictions.

4.	Thank you for your comments on the submission of images. We have removed the figures from the manuscript file and uploaded them as separate TIFF/EPS files.

**Responses from reviewer 1:**

1. Thank you for your insightful comments. We have revised the data presentation and interpretation for clarity and accuracy, and also performed a thorough proofreading to address grammatical errors.
2. We thank you for your suggestion regarding the title and have changed it to: "Post-Cesarean Section Infection Burden, Antimicrobial Resistance Pattern, and Associated Factors at ALERT Comprehensive Specialized Hospital, Addis Ababa, Ethiopia"
3. The affiliation of the principal investigator was changed to: "ALERT Comprehensive Specialized Hospital, Addis Ababa, Ethiopia"
4. The aim of the study was changed to: "This study aimed to assess the burden of bacterial infection after cesarean section, antimicrobial resistance patterns and related factors in mothers seeking postnatal care."
5. We added resistance and susceptibility rates for key bacterial isolates and included the number and percentage of MDR cases in the results section.
6. Bivariable and multivariable logistic regression was used to identify significant factors for infections after cesarean section, presenting only statistically significant results (p<0.05).
7. The introduction has been revised to improve focus, structure and clarity. It addresses the epidemiology and etiology of PSS infections and identifies existing knowledge gaps.
8. We note your comments on the complications of antibiotic-resistant infections and have revised our conclusions to reflect the burden observed in the study.

Introduction:

9. We thank you for your thoughtful feedback. We will restructure the introduction to provide greater clarity by starting with the epidemiology of PSS infections and clearly stating the research rationale.

**Methods:**

10. We have included a detailed description of our data collection methods to improve transparency and reproducibility.
11. We used the long form of the acronym (ALERT) as suggested.
12. We have provided a detailed description of data collection and processing to ensure accuracy and reliability.
13. The unnecessary subheadings have been removed.
14. The term “Gram-positive/Gram-negative” has been corrected to “Gram-positive/Gram-negative” throughout the document.

**Results:**

15. We have updated the terms for clarity and replaced "nulligravida" and "gravida" with the appropriate terms.
16. The phrase "risk factors" has been changed to "associated factors" throughout the document.
17. The bolded statements in the ‘clinical factors’ section were unintentional and have been standardized.
18. The binomial nomenclature issue has been corrected.
19. The citation of tables and figures has been standardized.
20. We will clearly present the calculation for the overall MDR in the 'Results' section.
21. The classification of antibiotics for MDR determination has been corrected and the tables will be reorganized for clarity.
22. The descriptions for tables and figures are revised to improve clarity and context.
23. The subheading in the discussion has been removed.

**Discussion**:

24. We will expand our discussion to provide a more thorough explanation of outcome variations.

**Conclusion:**

25.	The conclusion will be revised for specificity and clarity and necessary formatting adjustments will be made.

Reply to reviewer 2

Summary:

26. We appreciate your comment regarding the study design. We have clarified the term "cross-sectional study" in line with your suggestion.

27. The sentence regarding wound swabs has been revised for clarification: "Wound swabs were collected, processed for bacterial isolation and sensitivity testing was performed on the isolates."

28. The capitalization of “Gram” has been corrected.

**Introduction:**

29. We have used the acronym "SSI" according to its original definition throughout the manuscript.

30. The wording has been updated to "low- and middle-income countries"

31. The paragraphs defining SSI in the introduction have been merged for clarity.

32. The introduction was revised to define the knowledge gap and outline the objectives of the study.

**Material and methods:**

33. The study design has been updated to reflect the term "cross-sectional study"

34. We have provided a detailed description of steps taken to ensure the clinical relevance of isolated organisms.

35. A detailed description of the methods for confirming bacterial isolates at the species level included.

36. We acknowledge the importance of including MRSA identification and have incorporated the use of antibiotic disks for phenotypic identification.

**Thank you for your valuable feedback and support throughout this process!**
