# Supplementary material for: Post-caesarean section infection burden, antimicrobial resistance pattern, and associated factors at all Africa Leprosy Rehabilitation and Training Center Comprehensive Specialized Hospital, Addis Ababa, Ethiopia
Source: PLoS One. 2025 Jul 31;20(7):e0326935. doi: 10.1371/journal.pone.0326935 (PMC12312969; doi:10.1371/journal.pone.0326935)
Supplement: Attachment — Submitted filename: Response_to_reviewers_auresp_2.docx [file pone.0326935.s003.docx]

**Response to Reviewer Comments**

We sincerely appreciate the reviewers for their insightful and constructive feedback. Your time and effort in providing these comments have significantly enhanced the clarity and quality of our manuscript. We have addressed each comment thoroughly and apologize for any oversights in the previous version.

**Responses from Reviewer 1:**

- **Lines 44–45:** We have removed the italics for *coagulase-negative Staphylococcus* as suggested.
- **Line 52:** The phrasing has been corrected to “cesarean section (CSs)” as recommended.
- **Lines 77–78:** Revised to include: S. aureus, E. coli, Proteus mirabilis, and Staphylococcus epidermidis.
- **Lines 99–105:** We apologize for the lengthy explanation and have condensed this section for better focus.
- **Lines 36 & 114:** The capitalization of “Cross-sectional” has been corrected.
- **Line 107:** The subheading “Study area and setting” has been added.
- **Line 137:** We have inserted the subheading “Laboratory Methods.”
- **Around Line 153:** The subheading “Antimicrobial susceptibility testing” has been added as suggested.
- **Data Quality Assurance:** Thank you for identifying this area. We have revised this section to focus on relevant data quality assurance measures instead of lab procedures.
- **Line 195:** The subheading is now correctly titled “Data analysis and interpretation.”
- **Line 215:** We have inserted the subheading “Socio-demographic data” in the appropriate section.
- **Table 1:** The title has been revised to reflect the person, place, and time: “Socio-demographic factors of study participants at ALERT Comprehensive Specialized Hospital, Addis Ababa, Ethiopia, 2021.”
- **Line 236:** In response to your comment, we clarify that “clinical factors” refer to specific health conditions or characteristics observed in participants that may influence their overall health status or study outcomes.
- **Line 251:** The subheading “Surgical site infection” has been properly formatted.
- **Drug Names:** We apologize for previous misspellings. All drug names have been reviewed and corrected, such as changing “Ciprofloxine” to “Ciprofloxacin.”
- **Table Legends:** All abbreviations have been expanded in the table legends for clarity.
- **Line 297:** The MDR data has been revised for clarity, and the percentage calculation has been corrected. The notation 56/22 refers to a ratio of observed values, while (254.5%) indicates the percentage increase of multi-drug resistant cases compared to a baseline.
- **MDR Definition:** We have added an operational definition of MDR to clarify its usage in the results section.
- **Associated Factor Presentation:** The AOR, 95% CI, and P-values have been corrected and now follow the standard reporting format.
- **Table 5:** The table has been revised for completeness, ensuring all age groups are represented, and its structure has been improved.
- **Hypertension Analysis:** Thank you for your comment. Hypertension has now been included in the multivariate logistic regression analysis.
- **Discussion Section:** We appreciate your feedback and have thoroughly revised the discussion to ensure clear scientific reasoning and structured comparisons with relevant studies.

**Responses from Reviewer 2:**

- We are grateful for your positive feedback. All acronyms, including “CS” in the abstract, have been defined upon first use.
- We would like to extend our sincere appreciation to the reviewers and the editorial team. Your feedback has greatly improved the manuscript.
- Thank you for your insightful comment regarding the bivariate analysis. Hypertension was significantly associated with surgical site infections (COR = 12.59; p = 0.001), indicating its potential role as a risk factor. However, it was not included in the multivariate logistic regression due to specific considerations.
- These corrections have been made to enhance clarity and ensure that findings are reported according to accepted scientific conventions.
- We have also double-checked the consistency between the results in the tables and the text to eliminate discrepancies and improve readability.

Thank you once again for your invaluable feedback.
